# A Variable Temperature X-ray Diffraction Investigation of [PPN^+^][S_4_N_5_^−^]: Supramolecular Interactions Governing an Order/Disorder Transformation and the First High Resolution X-ray Structure of the Anion

**DOI:** 10.3390/molecules19021956

**Published:** 2014-02-12

**Authors:** René T. Boeré, Tracey L. Roemmele, Maria K. Krall

**Affiliations:** 1 Department of Chemistry and Biochemistry, University of Lethbridge, Lethbridge, AB T1K 3M4, Canada; E-Mails: roemtl@uleth.ca (T.L.R.); maria.ksiazek2@uleth.ca (M.K.K.); 2 The Canadian Centre for Advanced Fluorine Technologies, University of Lethbridge, Lethbridge, AB T1K 3M4, Canada

**Keywords:** sulfur-nitride, crystal structure, disorder modelling, DFT calculations

## Abstract

The title salt, triphenyl(*P,P,P*-triphenylphosphineimidato-*κN*)-phosphorus(1+) 1,3,5,7-tetrathia(1,5-*S*^IV^)-2,4,6,8,9-pentaazabicyclo[3.3.1]nona-1,4,6,7-tetraene(1−), CAS [48236-06-2], prepared by the literature method, is found by crystallography to be a 1:1 CH_3_CN solvate. Disorder exists for the N atoms of the anion. A VT crystal structure study was conducted at 100 K, 120 K, 140 K, 172 K, 200 K, 240 K and 280 K. The 100 K structure is superior, with only 10% of a second anion position oppositely-oriented w.r.t the diad axis of point group *2mm*. At 120 K, an adjacent two-site disorder is encountered, but at higher temperatures three-site disorder with both opposite and adjacent placements of S_4_N_5_^−^ ions is required w.r.t. the primary component. At 240 and especially 280 K, the anion nitrogen atoms appear fully scrambled amongst the six possible sites on the edges of an S_4_ tetrahedron with 83.3% occupancy for each. The PPN^+^ geometry does not show strong cation-cation interactions. However, there are numerous supramolecular contacts corresponding mostly to non-classical H-bonds between PPN^+^ ions and S_4_N_5_^−^ as well as CH_3_CN. The geometry of the anion is corroborated from B3LYP/6-311++G(3df) DFT calculations, and the infra-red spectrum was assigned with excellent agreement between experimental and calculated frequencies.

## 1. Introduction

**Figure 1:**
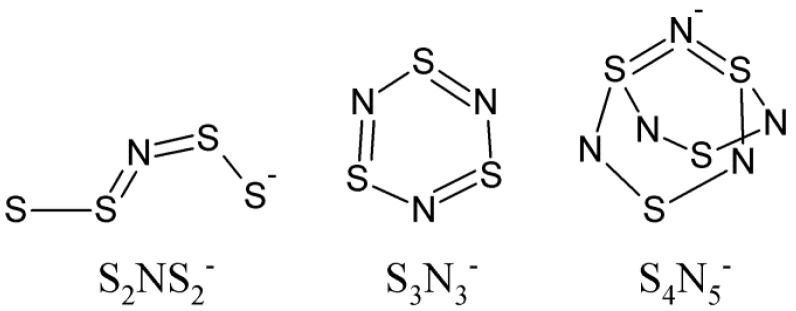
Structures of binary sulfur-nitrogen anions stabilized by PPN^+^.

Amongst the binary sulfur-nitrides, virtually all of which are endoergic, the sulfur-nitrogen anions are particularly noted for their instability with an enhanced tendency to decompose explosively [1,2,3]. The use of large cations was recognized early on in this chemistry as a mitigating factor in the stability of their salts [4]. In particular, the bis(triphenylphosphine)iminium ion, PPN^+^ [PPN^+^ = (Ph_3_P)_2_N^+^], was found to be particularly effective at stabilizing such anions [5,6,7,8,9,10,11,12,13,14,15,16,17]. In a short period in time, this cation was used to isolate thermally stable salts of S_4_N^−^, S_3_N_3_^−^ and S_4_N_5_^−^ (Figure 1) [5,6,7]. These stable, safe and thus *transportable* salts have enabled thorough investigations of properties such as ^14^N- and ^15^N- NMR spectroscopy [10,18,19,20], voltammetry and EPR spectroelectrochemistry [21,22,23,24], vibrational circular dichroism spectroscopy [25,26] and reactivity [8,27,28] including the generation of free radicals [29,30].

As part of our ongoing program of investigating the mechanisms of redox transformations of binary sulfur-nitrides [24,31] we have had occasion to make use of these fascinating PPN^+^ salts which are excellent for use in non-aqueous electrochemistry. They are very compatible with the *^n^*Bu_4_N^+^ cations that are components of the most common electrolytes employed in such solvents. In the course of this work, we also determined for the first time the crystal structure of [PPN^+^][S_3_N_3_^−^] and discovered that it crystalizes, when prepared by the literature method, as a 1:1 solvate with CH_3_OH [24]. The solvent is hydrogen-bonded to the anion within the cavities in the lattice created by the large and unwieldy PPN^+^ cations and the ensemble is otherwise well-behaved. By contrast, the smaller anions in crystals of [PPN^+^][SSNSS^−^] are positionally disordered [5,6,7]. We have now prepared [PPN^+^][S_4_N_5_^−^] and have discovered that its X-ray structure at 172 K shows positional disorder, as observed for the S_2_NS_2_^−^ salt, as well as solvent incorporation, as in the S_3_N_3_^−^ structure.

A review of the literature indicated that only two prior crystal structures have been reported that contain S_4_N_5_^−^. The first determination, in [*^n^*Bu_4_N^+^][S_4_N_5_^−^], is of quite low resolution, though it was of great importance at the time for establishing the connectivity in the anion [32]. The second is an unusual thallium salt that contains *both* S_3_N_3_^−^ and S_4_N_5_^−^ and, although more accurate, has the anions on symmetry-restricted sites [33]. Thus it seemed worthwhile to persist in trying to get a better structure from the PPN^+^ salt and this led us to undertake a thorough multi-temperature X-ray diffraction study the results of which are reported here. In so doing, we have detected a smooth order/disorder transformation with increasing temperature and developed a structural model that gives high-resolution atom coordinates and a geometry that agrees well with the results of DFT calculations using large basis sets. An analysis of the cation-anion interactions within the lattice show these to be primarily external charge-assisted non-classical hydrogen bonds that provide positional stabilization of the S_4_N_5_^−^ anion [34].

## 2. Results and Discussion

### 2.1. Sample Preparation and X-ray Diffraction Experiments

The title salt, triphenyl(*P,P,P*-triphenylphosphineimidato-*κN*)-phosphorus(1+) 1,3,5,7-tetrathia(1,5-*S*^IV^)-2,4,6,8,9-pentaazabicyclo[3.3.1]nona-1,4,6,7-tetraene(1−), CAS [48236-06-2], was first prepared from the methanolysis of bis(trimethylsilyl)sulfur diimide [35]. Soon afterwards, it was shown to be accessible by several routes, including the reactions of S_4_N_4_ with ammonia or metal azides [36,37,38,39]. We, however, prepared it by the currently recommended method [40]. The procedure involves first preparing the piperidine salt (which is fully characterized in the literature) followed by a cation exchange reaction and recrystallization from hot acetonitrile. *Please see the safety notice in the Experimental section.* In our hands, the procedure yielded the promised large yellow needles, from which suitably-sized blocks for X-ray diffraction could be obtained by cutting. Diffraction data sets were obtained at seven different temperatures, using three different crystals, as follows: a first crystal was measured at the facility’s default temperature of 172 K. When this was found to have significant anion disorder, a second crystal was determined at 120 K. Even at this temperature, a disorder which at first could not be modelled was encountered. Finally, a deliberate series of VT measurements were undertaken using a third crystal; first the crystal was carefully cooled to 100 K (the lower limit of our cooling system), a data set collected; then it was slowly warmed to 140 K, and the diffraction pattern measured again. Thereafter it was warmed to 200 K, 240 K and 280 K with data sets obtained at each point. The results indicate that full equilibration of the positional disorder is achieved by 280 K so there was no reason to continue to higher temperatures.

#### 2.1.1. The structure of [PPN^+^][S_4_N_5_^−^]∙CH_3_CN

**Figure 2:**
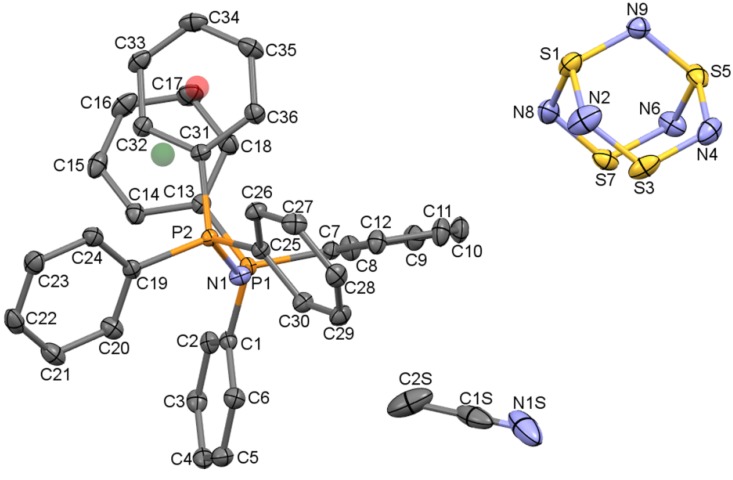
Displacement ellipsoids plot (50% probability) of [PPN^+^][S_4_N_5_^−^]∙CH_3_CN at 100 K from the X-ray diffraction structure. H-atoms and the minor (10%) disorder component of the anion are omitted. Green and red spheres are ring centroids for PPN^+^ phenyl rings.

The structures solved in the well-behaved space group *P*2_1_/*c* and contain discrete PPN^+^ cations (see Section 2.1.3) and S_4_N_5_^−^. In addition, a previously unreported acetonitrile of solvation is present in the lattice with full occupancy and in a 1:1 ratio with the salt components (Figure 2). It quickly became apparent that the dataset collected at 100 K was superior to the others and this was taken as a starting point for the structure analysis of the anion (see Section 2.1.2). In fact, all the datasets are found to contain the PPN^+^ and acetonitrile solvent in the same spatial locations. There is some evidence for minor site dislocation of the solvent from the shapes of the displacement ellipsoids, but since this was not found to perturb the anion, these minor deviations have been ignored as CH_3_CN is a structurally well characterized species, unlike S_4_N_5_^−^. Furthermore, it quickly became apparent that the four heavy sulfur atoms of the anion are also invariantly located in the lattice, so that the positional disorder concerns mainly the five nitrogen atoms of the anion. Moreover, except at the highest temperatures (240, 280 K) where complete randomization of the N sites occurs, the location of the dominant component of S_4_N_5_^−^ in each derived crystal structure is the same, indicative of some significant ordering effects in the lattice.

#### 2.1.2. S_4_N_5_^−^ Disorder as a Function of Temperature

**Figure 3:**
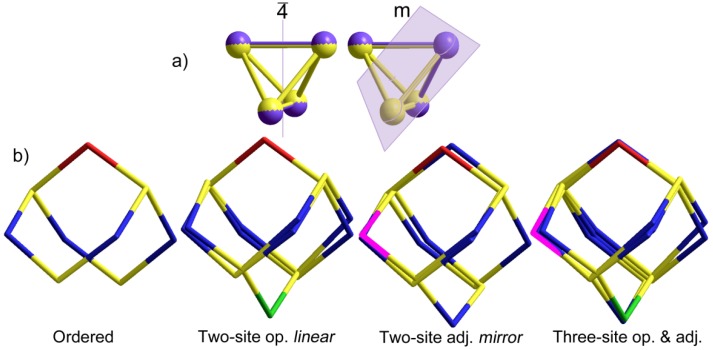
(**a**) Pairs of capped tetrahedra arranged with the two unique (N9) caps indicated by purple *opposite* (left) or *adjacent* (right), showing the locations of the resultant symmetry elements; (**b**) S_4_N_5_^−^ ions in the four orientations discussed in the text. To aid in identification, the unique capping nitrogen atom has been colored red for the reference orientation, green for the one added opposite and magenta for the one added adjacent.

The cage structure of S_4_N_5_^−^ (Figure 2) is often described as a tetrahedron of S atoms symmetrically bridged on five of the six tetrahedron edges by N atoms, with very little distortion from the ideal geometry imposed by the capping atoms. Indeed, in the experimental geometry of the best defined cluster (main component of the 100 K structure, see below), the six S–S distances range from a low of 2.695(1) to a high of 2.769(2) Å, less than 3% variation; treating this as an S_4_ tetrahedron thus seems very reasonable. For combinations of two penta-bridged tetrahedra, the second orientation may be opposite the unique bridge (in symmetry terms, aligned with the 2-fold axis of the *2mm* point group of S_4_N_5_^−^) which results in a system with *linear* symmetry (in the limit of equal populations, this axis becomes 4, Figure 3a). Note that the symmetry is not tetrahedral since the populations of N sites have ratios (from the “top” down) of 0.5:1:1:1:1:0.5. Alternatively, the second orientation may be adjacent to the unique bridge. The resulting combination has *mirror* symmetry (Figure 3a). There are four such possible adjacent combinations, each one resulting in populations of 1:1:1:1:0.5:0.5, that is, one of the four beltline sites has single occupancy (Figure 3b). A further possibility needs consideration, wherein both the opposite and adjacent sites get occupied. Even with equal proportions of the components, the site populations for the six nitrogen positions can range from a low of (2 × 0.5 + 4 × 1 = 5) for two disorder components, (3 × 0.333 + 3 × 1 = 5) for three, *etc*. up to complete equivalence at (6 × 0.833 = 5). With partial occupancies as occurs in the experimental disorders, the possible populations at individual N sites quickly become very diverse.

**Figure 4:**
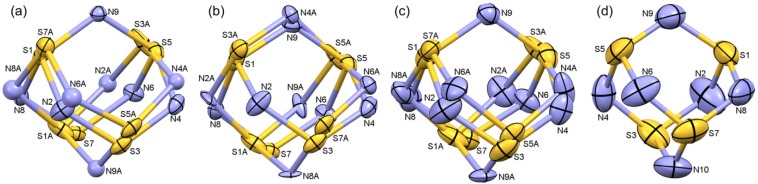
Refined disorder models of the anion S_4_N_5_^−^ at: (**a**) 100 K; (**b**) 120 K; (**c**) 172 K; (**d**) 280 K. Displacement ellipsoids are at 30%. Atom labels reflect the main occupancies.

The models that were constructed from the diffraction intensity data fall into four classes. For crystal 3 at 100 K, the dominant orientation has a refined occupancy of 90%. A difference Fourier map of the residual electron density (e.d.) shows a well-defined residue corresponding to the opposed two-site disorder model with linear symmetry. This model refines fairly well, but with only 10% of the e.d., further nuances to the disorder are difficult to detect (Figure 4a). Gratifyingly, the refinement based on this model suffices so that the 100 K structure provides excellent data for the structure of S_4_N_5_^−^ from the main component (Table 1). For crystal 2 at 120 K, the main component has 85% occupancy, and the difference map is a good fit to a two-site disorder with mirror symmetry. This model refines very well as an adjacent model, and with more e.d. available even the disordered N atoms can be refined reasonably with anisotropic displacements (Figure 4b). Here, the geometry of the main component (Table 1) is also quite well-defined. 

**Table 1:** Selected Interatomic Distances in S_4_N_5_^−^ Structures from Experiments and Computation.

Parameter, Å or °	100 K *^a^*	120 K *^a^*	172 K *^a^*	Flues *et al. ^b^*	Martan *et al. ^c^*	DFT *^d^*
S3∙∙∙S7	2.695(1)	2.697(2)	2.724(6)	2.71(1)	2.657(10)	2.8417
S1∙∙∙S5	2.769(1)	2.763(3)	2.760(6)	2.75(1)	2.795	2.7913
S1–N2	1.657(2)	1.657(3)	1.690(8)	1.62(2)	1.67(1)	1.6667
S1–N8	1.666(2)	1.668(3)	1.655(6)	1.65(2)	1.67(1) *^g^*	1.6688
S5–N4	1.677(2)	1.676(4)	1.703(8)	1.63(2)	1.67(2)	1.6687
S5–N6	1.6710(17)	1.670(3)	1.683(8)	1.66(2)	1.67(2) *^g^*	1.6667
S3–N2	1.614(2)	1.619(3)	1.605(9)	1.61(2)	1.61(1)	1.6171
S3–N4	1.604(2)	1.599(4)	1.574(8)	1.63(2)	1.61(1)	1.6164
S7–N6	1.6080(17)	1.608(3)	1.581(6)	1.60(2)	1.61(1) *^g^*	1.6172
S7–N8	1.6170(18)	1.615(3)	1.610(6)	1.57(2)	1.61(1) *^g^*	1.6164
S1–N9	1.6450(17	1.640(4)	1.664(5)	1.68(2)	1.64(2)	1.6484
S5–N9	1.6446(19)	1.621(5)	1.665(5)	1.64(2)	1.64(2)	1.6485
P1–N1	1.5855(11)	1.5838(14)	1.5803(12)	—	—	—
P2–N1	1.5824(11)	1.5794(14)	1.5788(13)	—	—	—
S1–N2–S3	114.15(12)	114.06(15)	115.1(5)	113(1)	113.2(8)	114.80
N2–S3–N4	115.20(12)	114.32(17)	112.9(4)	114(1)	113.5(8)	114.21
S3–N4–S5	113.03(11)	113.3(2)	113.8(6)	114(1)	113.7(9)	114.82
N4–S5–N6	99.33(10)	99.14(18)	100.0(4)	100(1)	101(1)	101.69
S5–N6–S7	113.57(10)	113.51(19)	114.5(4)	115(1)	113.7(9) *^g^*	114.80
N6–S7–N8	113.82(9)	113.83(16)	113.6(4)	114(1)	113.5(8) *^g^*	114.21
S7–N8–S1	113.13(10)	112.76(18)	112.7(4)	114(1)	113.2(8) *^g^*	114.81
N8–S1–N2	99.65(11)	100.91(17)	101.3(4)	101(1)	100(1) *^g^*	101.68
N2–S1–N9	109.47(11)	108.7(3)	107.6(4	110(1)	117(1)	108.49
N8–S1–N9	109.34(9)	108.6(2)	111.1(3)	111(1)	108.3(6) *^g^*	108.41
N4–S5–N9	109.62(11)	110.0(3)	108.2(4)	110(1)	107.7(6)	108.41
N6–S5–N9	109.52(11)	108.8(2)	110.3(3)	109(1)	107.7(6) *^g^*	108.49
S1–N9–S5	114.68(10)	115.8(3)	112.1(2)	112(1)	117(1)	115.70
∠S_1_N_8_N_6_S_5_:S_1_N_9_S_5_^*e*^	49.51(9)	50.7(4)	47.6(2)	—	50.2	50.86
∠S_1_N_2_N_4_S_5_:S_1_N_9_S_5_ *^e^*	50.01(9)	49.3(3)	53.1(3)	—	50.2 *^g^*	50.86
P1–N1–P2	137.81(7)	138.16(10)	138.98(9)	—	—	—
Ct^1^–P1–P2–Ct^2 *f*^	−32.99	−33.05	−33.33	—	—	—

*^a^* This work; taken from the main components of the disorder models; *^b^* [*^n^*Bu_4_N][S_4_N_5_^−^], ref. [32]; *^c^* Tl_2_[S_4_N_5_^−^][S_3_N_3_^−^], ref. [33]; *^d^* B3LYP/6-311++G(3df) symmetry unrestricted; *^e^* “Envelope” dihedral; *^f^* The ring offset angle, *i.e.*, torsion angle for centroid-P1-P2-centroid. See Section 2.1.3.; *^g^* Structure has *m* site symmetry.

For higher temperatures, neither of these two models on their own suffices. For example, for crystal 1 at 172 K the difference map appears to fit to the two-site opposite orientation, but refinement leads to an unacceptably large displacement parameter for “N6A” (Figure 4c). This indicates the presence of a three-site disorder model that combines the opposite and adjacent occupancies. It was not possible to identify three separate sets of *x,y,z* coordinates for this model; instead the “opposite” two-site model was modified by simply adjusting the occupancy factors for the N sites affected by populating the adjacent orientation (interestingly, this is the same “adjacent” location that is identified in the 120 K structure). An acceptable model was obtained when the populations reached 60:20:20 ratios for main, opposite and adjacent orientations (Figure 4c). Similar three-site models fit very well for crystal 3 at higher temperatures, namely at 140 K (70:15:15) and 200 K (57:25:18). Finally, above 200 K the electron density distribution in these crystals could not be distinguished accurately, so that full randomization of the occupancy of all six N sites was employed using only a single set of coordinates to model crystal 3 at 240 K and 280 K (Figure 4d). While this leads to an acceptable refinement without significant residuals and with well-shaped displacement ellipsoids, such a model wipes out any distinction amongst the different S to N bonds, and must be judged chemically unacceptable; thus the higher temperature refinements are not useful for establishing the *structure* of S_4_N_5_^−^ (but see Section 2.1.4). It is also instructive to consider the 172 K structure more critically: a full refinement is possible for this structure using two sets of S and N coordinates and the result appears to be crystallographically beautiful, without significant residuals and with very reasonable displacement ellipsoids for all sites. Nevertheless, the *geometry* of the structure (Table 1) has been significantly compromised through the third site occupancy, resulting in a much greater scatter in what should be symmetry equivalent bond distances (Table 2). Thus, the 172 K structure is inferior for establishing the structure of S_4_N_5_^−^, improving only slightly on that of [*^n^*Bu_4_N^+^][S_4_N_5_^−^] [32].

**Table 2:** Averaged Interatomic Distances in S_4_N_5_^−^ from Experiments and Computation. *^a^*

Parameter, *^b^* Å or °	100 K	120 K	172 K	Flues *et al. ^c^*	Martan *et al. ^d^*	DFT *^e^*
S3∙∙∙S7	*2.695(1)*	*2.697(2)*	*2.724(6)*	*2.71(1)*	*2.667(10)*	**2.8427**
S3–N2	1.611(6)	1.610(9)	1.593(18)	1.60(3)	1.61(1)	1.6166
S1–N2	1.668(8)	1.668(8)	1.68(2)	1.640(18)	1.67(1)	1.6675
S1–N9	1.6448(3)	1.631(13)	1.665(1)	1.66(3)	1.64(1)	1.6486
S1∙∙∙S5	*2.769(1)*	*2.763(3)*	*2.760(6)*	*2.75(1)*	*2.795*	2.7914
S1–N2–S3	113.5(5)	113.4(5)	114.0(10)	113.5(10)	113(2)	114.81
N2–S3–N4	114.5(10)	114.4(3)	113.3(5)	113.5(10)	113.5(10)	114.21
N2–S1–N8	99. 5(2)	100.0(13)	100.7(9)	100.5(10)	100(1)	101.69
N2–S1–N9	109.49(12)	109.0(7)	109.3(17)	110.1(10)	110(3)	108.45
S1–N9–S5	*114.68(10)*	*115.8(3)*	*112.1(2)*	*112(1)*	*117*	115.70

*^a^* Errors here are std. dev. of the samples; *italic* single val; *^b^* Symmetry-equivalent values in point group *2mm* specified by the first exemplars (see Figure 2 for numbering scheme); *^c^* [*^n^*Bu_4_N][S_4_N_5_^−^], ref. [32]; *^d^* Tl_2_[S_4_N_5_^−^][S_3_N_3_^−^], ref. [33]. *^e^* Calc. symmetry-restricted to *2mm*.

Table 2 presents a summary wherein symmetry equivalent distances and angles (in point group *2mm*) have been averaged from the data in Table 1. The 100 K structure has systematically lower standard deviations for the average values, and deviates from the DFT calculated values by on average half as much as the higher temperature structures, and on average one third as much as [*^n^*Bu_4_N^+^][S_4_N_5_^−^] [32]. An interesting outcome of this study is the confirmation it provides for the basic correctness of the structure of the anion as determined in the mixed salt Tl_2_[N_3_S_3_][N_4_S_5_] many years ago [33]. This compound does share the attribute of using a large cation and does not suffer from positional disorder; indeed the anion crystallizes with *m* site symmetry. On the other hand, this compound has apparently never been used for property measurements and it is not known to be safe against detonation (the short chemical note in the only paper about this compound mentions only that it burns vigorously in a flame [33].)

From the 100 K data, the S3–N2 bond type (within the lower N=S=N units) is 8.5% shorter than the sums of S and N covalent radii (a “single bond”) using the 2008 revaluated covalent radii values [41], and is also 0.9% shorter than the average S–N bond distance of 1.625(7) Å in S_4_N_4_ according to a recent low-temperature crystal structure determination [42]. The S1–N2 bond type, that is the bond to the sulfur atoms that bear the “added” N^−^ ion, is 5.2% shorter than a single bond, but 2.6% *longer* than the average bond in S_4_N_4_ [42], whereas the S1–N9 bond type, to the capping N, is 6.5% shorter than a single bond and 1.2% shorter than S_4_N_4_ bonds. Both S∙∙∙S transannular distances are longer than in S_4_N_4_, by 6.8% for S3∙∙∙S7 and 3.9% for S1∙∙∙S5, but these values are still less than the sums of their v.d. Waals’ radii (∑*r*_vdW_) by 17.3 and 19.6%, respectively [43,44].

#### 2.1.3. On the Structure of PPN^+^

The PPN^+^ ion remains very popular, particularly for coordination compounds. Some 1,373 structures are listed in version 5.34 (May 2013 release) of the Cambridge Structural Database [45], up from 752 when Lewis and Dance published their retrospective analysis of the crystal packing properties of this cation [46]. PPN^+^ ions crystallize in a wide variety of geometries; for example, the P–N–P angle can range from 130–180°; the value of 137.81(7)° in the 100 K structure of [PPN^+^][S_4_N_5_^−^]∙CH_3_CN is close to the mean value of 143.1° [46]. Some structures have one phenyl ring on each PPh_3_ group close to being eclipsed, the so-called offset face-to-face (**off**) conformation [47]. The cation in [PPN^+^][S_4_N_5_^−^]∙CH_3_CN has a centroid-to-centroid separation (Figure 1) of 3.82 Å, and the offset angle (defined as the torsion from centroid-P1-P2-centroid) is 33.1°, indicating a weak **off** interaction. Lewis and Dance have identified particularly strong *cation-cation* interactions when the shortest P∙∙∙P separation in different ions is <7.25 Å [46]. In [PPN^+^][S_4_N_5_^−^]∙CH_3_CN, the shortest such separation is 7.9407(6), indicating that strong cation-cation forces are probably absent; hence it is appropriate to focus on the cation-anion interactions.

#### 2.1.4. Supramolecular Interactions in the [PPN^+^][S_4_N_5_^−^] Lattice

**Figure 5:**
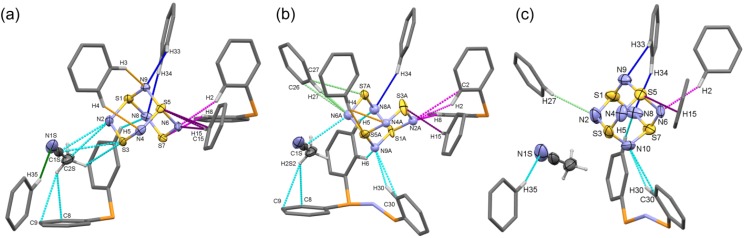
Intermolecular interactions < ∑*r*_vdW_ in various crystal lattices of [PPN^+^][S_4_N_5_^−^]. (**a**) Contacts to the main component at 100 K; (**b**) Contacts to the minor component at 100 K. (**c**) Contacts at 280 K to a composite structure. The color-coding of the interactions is the same across the three pictures.

The combined results of these models developed for crystals with different thermal histories suggests that the lattice in [PPN^+^][S_4_N_5_^−^] crystals provides a substantial orienting effect on the almost globular shape of the anion; apparently this is opposed by thermal energy that smoothly leads to the randomization of the occupancies already below RT. An analysis has been undertaken of the intermolecular interactions in the two limiting data sets, the 100 K structure in its major and minor occupancies, and the 280 K structure with its averaged environment. A diagram showing the interactions which are < ∑*r*_vdW_ for these three situations is provided in Figure 5. The colour coding is by the symmetry codes of the ions, with cyan used to indicate interactions within the same asymmetric unit as the S_4_N_5_^−^ ion, with the same colours identifying the same symmetry relationships in each picture. The numerical data corresponding to these pictures is provided in Table 3 for the more important contacts which are conservatively restricted to ≤ (∑*r*_vdW_ − 0.1 Å).

**Table 3:** Intermolecular contacts in [PPN^+^][S_4_N_5_^−^] lattices that have *d* ≤ (∑*r*_vdW_ − 0.1 Å).

Component	Atom1	Atom2	Symm. op. 1	Symm. op. 2	Length	*d*−∑*r*_vdW_	*d*−Wk	Type
100K Main	N6	H2	x,y,z	1−x,1−y,−z	2.479	−0.271	−0.051	n.c. H-bond
100K Main	N6	H15	x,y,z	x,1+y,z	2.481	−0.269	−0.049	n.c. H-bond
100K Main	N1S	H35	1−x,1/2+y,1/2−z	x,1.5−y,1/2+z	2.531	−0.219	0.001	n.c. H-bond
100K Main	S5	H15	x,y,z	x,1+y,z	2.796	−0.204	−0.104	n.c. H-bond
100K Main	S7	H8	x,y,z	1−x,1−y,−z	2.865	−0.135	−0.035	n.c. H-bond
100K Main	S5	C15	x,y,z	x,1+y,z	3.376	−0.124	n/a	n.c. H-bond
100K Main	N2	C2S	x,y,z	1−x,1/2+y,1/2−z	3.143	−0.107	n/a	N≡C carbon π
100K Minor	N6A	H27	x,y,z	−x,1/2+y,1/2−z	2.35	−0.400	−0.180	n.c. H-bond
100K Minor	N9A	H30	x,y,z	1−x,1/2+y,1/2−z	2.365	−0.385	−0.165	n.c. H-bond
100K Minor	N2A	H2	x,y,z	1−x,1−y,−z	2.432	−0.318	−0.098	n.c. H-bond
100K Minor	N6A	C27	x,y,z	−x,1/2+y,1/2−z	3.022	−0.228	n/a	n.c. H-bond
100K Minor	N8A	H5	x,y,z	1−x,1/2+y,1/2−z	2.524	−0.226	−0.006	n.c. H-bond
100K Minor	S5A	H4	x,y,z	−1+x,1+y,z	2.801	−0.199	−0.099	n.c. H-bond
100K Minor	S1A	H6	x,y,z	1−x,1/2+y,1/2−z	2.807	−0.193	−0.093	n.c. H-bond
100K Minor	N4A	H4	x,y,z	−1+x,1+y,z	2.62	−0.130	0.090	n.c. H-bond
100K Minor	N6A	C1S	x,y,z	1−x,1/2+y,1/2−z	3.126	−0.124	n/a	N≡C carbon π
100K Minor	S3A	H15	x,y,z	x,1+y,z	2.893	−0.107	−0.007	n.c. H-bond
100K Minor	N9A	C30	x,y,z	1−x,1/2+y,1/2−z	3.15	−0.100	n/a	n.c. H-bond
280K Single	N10	H30	x,y,z	1−x,1/2+y,1/2−z	2.511	−0.239	−0.019	n.c. H-bond
280K Single	N6	H2	x,y,z	1−x,1/2+y,1/2−z	2.531	−0.219	0.001	n.c. H-bond
280K Single	N8	H5	x,y,z	1−x,1/2+y,1/2−z	2.617	−0.133	0.087	n.c. H-bond
280K Single	N1S	H35	1−x,1/2+y,1/2−z	x,1.5−y,1/2+z	2.625	−0.125	0.095	n.c. H-bond

This analysis confirms the assertion made above that the location of the anion and CH_3_CN solvent is well-defined in the PPN^+^ cavities at all temperatures. Thus, the atom site denoted “N10” at 280 K is precisely the alternate capping N position N9A in the minor component at 100 K, and when the adjacent site is populated, it appears to be in only one of the four possible locations. Almost all the interactions correspond to external charge-assisted non-classical hydrogen bonding and the strongest contacts persist over the 180 K range (N6∙∙∙H2, N1S∙∙∙H35 for the solvent, N9A/N10∙∙∙H30, N8∙∙∙H5, S5∙∙∙H15) [34,48,49,50,51]. On the other hand, the strengths of all the contacts decrease with increasing temperature, thereby reducing the number that is ≤ (∑*r*_vdW_ − 0.1 Å) from 7 for the main and 11 for the minor component at 100 K to four at 280 K. On balance, it does not appear that there is any single dominant interaction that results in the selection of the favored orientation of the anion. Rather, it seems as if a combination of non-classical H-bonds and steric interactions provides a slightly better fit for the main orientation compared to the ‘opposite’ or ‘adjacent’ alternatives. The location of the solvent in the lattice may well play a role in this although, based on distances, anion-solvent interactions are quite weak. Thus, the site-selectivity for the S_4_N_5_^−^ anion in this salt seems to be similar to that encountered for substrates and substrate/co-factor combinations at active sites of many enzymes in biology: that is, a subtle balance of forces results in weak, but sufficient, ordering of the substrate.

**Figure 6:**
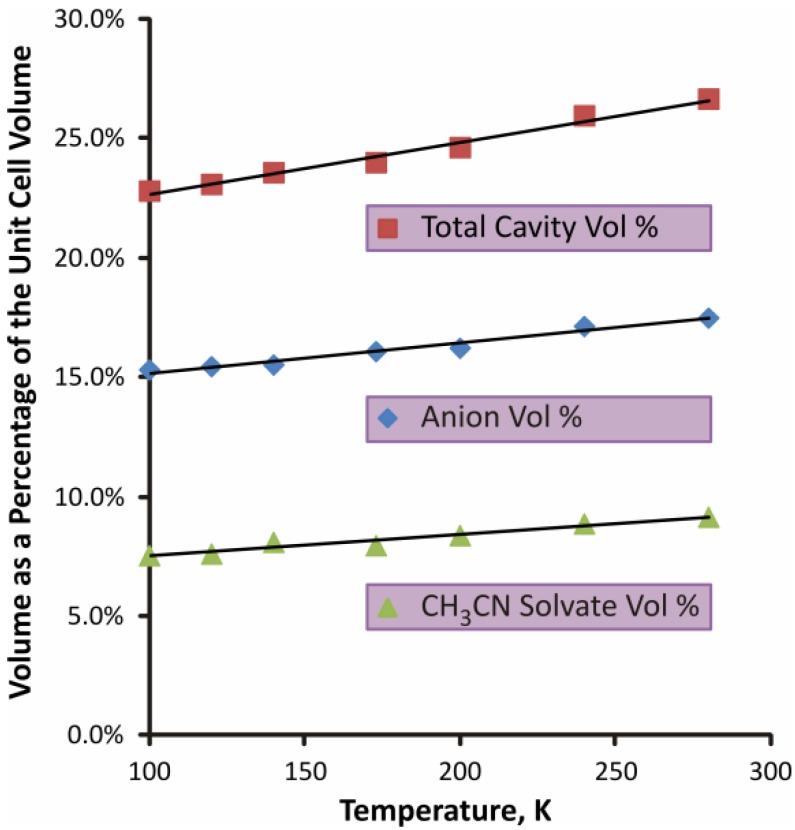
Graphs of the changes in percentages of the overall unit cell volume available to the anion, the solvent, and for both combined, with increasing temperature.

The longer distances for the “short contacts” in the structure at 280 K matches well with the overall increase in the volume that the anion and solvent are able to occupy within the lattice. By using a void analysis calculation that is available in Mercury, release 3.1.1 [52], it can be shown that the volume of the cavities created between the PPN^+^ phenyl rings rises faster than the overall increase in unit cell volume as the crystal thermally expands from 100 K to 280 K. Thus, while the overall unit cell volume increases by 4.7%, the volume available to the anion increases by 19.5% over this temperature range. This can be shown graphically in Figure 6, where the percentages of the unit cell volume available to the anion, the solvate molecule and the overall cavity size are graphed *relative* to the cell volumes at each temperature; all the curves are reasonably linear with positive slopes.

The combination of this thermal volume analysis and the evidence for positional regularity of the “S_4_N_6_” framework could fit for a thermally activated “rotational jump” mechanism wherein S_4_N_5_^−^ can exchange its orientation but not its framework location, whether that location is due to the larger size of the sulfur atoms or is caused by the non-classical H-bonding to the nitrogen atoms. Certainly, all the orientations of the anion identified in this study are accessible by such a rotational mechanism.

**Scheme 1:**
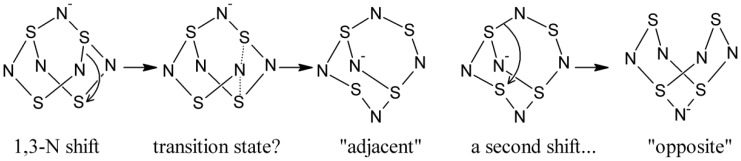
Interconversion of S_4_N_5_^−^ positions through 1,3-N shifts.

However, another possibility needs to be considered (Scheme 1) since it is now well-established that 1,3-nitrogen shift reactions are possible for many sulfur-nitrogen rings and cages [2]. This was first discovered from the scrambling of ^15^N labels introduced into PhCN_5_S_3_ [53,54] and also found as the operative mechanism for the transformation of this same type of cage molecule into trithiatetrazocines under nucleophilic attack [55]. A very similar 1,3-N shift process is believed to operate in the conversion of the anion S_4_N_4_^−^ into S_3_N_3_^−^ and NS^•^ after chemical or electrochemical reduction of S_4_N_4_ [24]. However, the possibility that such sigmatropic rearrangements could operate was first proposed by Bartetzko and Gleiter specifically for the binary sulfur nitrogen cage compounds S_4_N_5_^+^ and S_4_N_5_^−^ [56]. S_4_N_5_^+^ is isoelectronic with PhCN_5_S_3_ through the replacement of S^+^ by an RC group, so by analogy to the latter, it is highly likely to be fluxional. While it has been suggested that for such a shift to also operate in S_4_N_5_^−^ is less likely because the latter possesses a transannular S∙∙∙S contact (*i.e.*, between S3 and S7 in our numbering scheme) [54], this has never been proven. As shown in Scheme 1, a single 1,3-N shift could convert S_4_N_5_^−^ from the main orientation into the “adjacent” within the lattice. Two sequential bond shifts are required to convert it to the opposite orientation.

### 2.2. Density Functional Theory Study of the Geometry of S_4_N_5_^−^ in the Gas Phase

While it is often thought that the PPN^+^ cation provides a minimally interactive environment and thus less likely to distort the structure of sensitive anions, the requirement in the case of S_4_N_5_^−^ to go to extremely low temperatures to obtain an ordered structure provides at least the potential that the LT environment results in a distortion of the structure. Therefore it is important to verify the experimental geometry and for this purpose we report the results of high-level density functional theory calculations on the gas phase S_4_N_5_^−^ anion at 0 K. Thus the geometry of the anion was successfully optimized using B3LYP/6-311++G(3df), a large triple ζ basis set. Frequency calculations confirmed that the geometries are at least local minima. The metrical parameters of this calculation are provided in Table 1 in parallel with the results from the VT-diffraction study. The agreement between the 100 K X-ray structure and this DFT geometry is extremely good (Table 2). The one caveat concerns the length of the non-bonded S3∙∙∙S7 contact, which is 0.15 Å (~5%) greater in the DFT results, especially in view of the good agreement on all other metrics. Can this come about from the lattice “pinching” the anion so that the structure determination is compromised? In fact, we think it much more likely that it is the DFT result that is incorrect for this particular parameter, since DFT requires the addition of dispersion functions in order to correctly model the diffuse π*-π* transannular “bonding” between parallel NSN units (*i.e.*, from N2,S3,N4 to N6,S7,N8).

### 2.3. Confirmation and Assignment of the Bands in Infra-red Spectra of S_4_N_5_^−^

**Figure 7:**
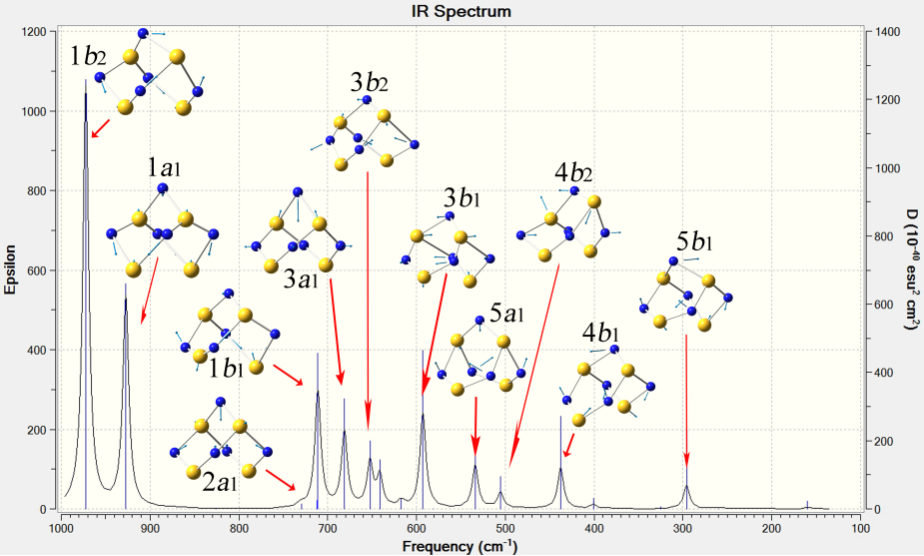
The DFT computed IR spectrum for an S_4_N_5_^−^ anion, showing the principal vibrational modes which have appreciable intensity; frequencies here are *not* scaled.

The electronic structure of S_4_N_5_^−^ has apparently not been revisited since early extended Hückel [56,57], MNDO [58] and HFS-SCF treatments [59]. The B3LYP/6-311++G(3df) frequency calculation undertaken for this work provides an opportunity to confirm the vibrational spectra of this fundamental anion and provide tentative assignments of the bands (Table 4, Figure 7). Infra-red spectroscopy is important for binary sulfur nitrides which lack suitable NMR nuclides convenient for identification or monitoring purities of samples. It is therefore fortunate that the IR bands are generally very characteristic of a given species, sufficient to allow for both identification and detecting impurities (especially if those are *different* binary sulfur nitrides.) There is in general very good agreement between the gas-phase calculated frequencies (in cm^−1^, which have been corrected by the appropriate scale factor of 0.967 ± 0.019 [60]), and several reports for experimental spectra obtained on solids. Several bands, namely 2*a*_1_, 2*b*_2_, 6*a*_1_ and 3*a*_2_, are observed despite predicted oscillator strengths close to or equal zero; these must gain intensity in condensed phases from reduced symmetry through inter-molecular interactions. The worst agreement between calculated and observed frequencies is for 2*a*_1_, 2*b*_2_ and 3*b*_1_.

**Table 4:** Calculated and experimental infra-red spectral data for M^+^[S_4_N_5_^−^].

Mode	DFT calc. cm^−1^ *^a^*	Calc. rel. intensity *^a^*	Ave. exptl. cm^−1^ *^b^*	PPN^+^ *^c^* nujol/CsI	Li^+^ *^d^* nujol/CsI	NH_4_^+^ *^d^* nujol/CsI	PipNH_2_^+^ *^e^* nujol/CsI	Na^+^ *^f^* KBr
1*b*_2_	940	1.00	946, vs	955, s	940, vs	940, vs	945, vs	950, vs
1*a*_1_	897	0.50	906, vs	916, s	910, vs	910, vs	910, vs	885, sh
1*a*_2_	798	0.00	not obs.	—	—	—	—	—
2*a*_1_	706	*0.01*	744, m	757, m	750, m	735, s	735, s	745, s
2*b*_2_	688	*0.01*	731, m *^g^*	747, m	—	725, s	725, s	725, w
1*b*_1_	688	0.27	693, s *^g^*	—	700,s s	685, s	685, s	700, s
3­*a*_1_	659	0.18	667, m	666, w	670, s	663, s	670, s	665, vs
3*b*_2_	631	0.11	645, w	647, w	—	—	652, m	635, w
2*b*_1_	620	0.07	623, w	—	625, w	620, w	625, m	—
4*a*_1_	597	*0.02*	not obs.	—	—	—	—	—
3*b*_1_	573	0.23	600, s	598, m	600, s	600, s	600, s	600, vs
2*a*_2_	562	0.00	not obs.	—	—	—	—	—
5*a*_1_	516	0.10	531, s	—	530, s	530, s	532, s	530, vs
4*b*_2_	489	0.04	505, s	—	510, m	500, s	503, s	505, s
4*b*_1_	423	0.10	436, s	428, m	440, s	435, m	435, s	440, vs
*6a_1_*	*387*	*0.01*	406, m	—	410, m	405, m	400, m	410, s
3*a*_2_	322	*0.00*	338, w	—	345, w	335, w	335, w	335, w
5*b*_2_	314	0.00	not obs.	—	—	—	—	—
5*b*_1_	286	0.06	304, w	300, vw	310, m	300, w	305, m	305, m
4*a*_2_	252	0.00	not obs.	—	—	—	—	
7*a*_1_	155	0.00	not obs.	*Beyond range of experiments.*				

*^a^* B3LYP/6-311++G(3df); freq. scaled by 0.967; *^b^* Compiled from the five salts; *^c^* Ref. [6]; *^d^* Ref. [39]; *^e^* The piperidyl cation, Ref. [39]; *^f^* Ref. [35]; *^g^* These two assignments are based on intensities only.

## 3. Experimental

*CAUTION: salts of S_4_N_5_^−^ and other binary sulfur-nitrides are notorious contact explosives. Any attempts to prepare or handle them must only be undertaken by appropriately qualified personnel, properly supervised, and taking all necessary precautionary measures*.

Samples of [PPN^+^][S_4_N_5_^−^]∙CH_3_CN were prepared according to the literature method [40]. Crystals suitable for X-ray diffraction were grown, as recommended, from the slow cooling of an acetonitrile solution that was saturated when warm. Large needles were selected under a microscope and cut into suitably sized blocks, taking care to ensure no large stresses are introduced by visualizing before and after handling with transmitted polarized light and crossed polarizers. Selected crystals were mounted on the ends of thin capillaries either in Paratone™ or immobilized in epoxy glue, followed by careful cooling on the goniometer head using the Kryoflex cooling accessory. Data were collected on a Bruker ApexII CCD area-detector diffractometer with ϕ and ω scans using Mo *K*_a_ radiation (λ = 0.71073 Å). A hemisphere of data was collected with the aid of APEX2 control software; data were refined and reduced with SAINT-Plus and a semi-empirical absorption correction from equivalents was applied to the data using SADABS (APEX2, SAINT-Plus and SADABS. 2008, Bruker AXS Inc., Madison, WI, USA). Structure solution by direct methods using SHELXS-2013 was followed by full-matrix least squares refinement with SHELXL-2013 [61]. The composition of the crystals confirmed by the structure determination corresponds to a moiety formula of [C_38_H_33_NP_2_][N_5_S_4_]∙C_2_H_3_N, with a FW of 777.89 amu. The crystal system is monoclinic and the space group is *P*2_1_/*c* at all temperatures with *Z* = 4. Data and parameters specific to the different crystals measured and the different data collection temperatures are presented in Table 5. CCDC 980863 for 100 K, 980864 for 120 K, 980865 for 140 K, 980866 for 172 K, 980867 for 200 K, 980868 for 240 K and 980869 for 280 K contain the supplementary crystallographic data for this paper. These data can be obtained free of charge at http://www.ccdccam.ac.uk/const/retrieving.html or from the Cambridge Crystallographic Data Centre (CCDC), 12 Union Road, Cambridge CB2 1EZ, UK; fax: +44(0)1223-336033 or e-mail: deposit@ccdc.cam.ac.uk.

**Table 5:** Crystal data and structure refinement parameters for S_4_N_5_^−^ at different temperatures.

Parameter	100 K	120 K	140 K	172 K	200 K	240 K	280 K
*a*, Å	10.8717(5)	10.8904(6)	10.9331(5)	10.9878(6)	11.0218(5)	11.0664(7)	11.131(18)
*b*, Å	17.7722(8)	17.7756(10)	17.8025(8)	17.8189(10)	17.8463(8)	17.8745(11)	17.92(3)
*c*, Å	18.8902(8)	18.8948(11)	18.9084(9)	18.9099(11)	18.9487(9)	18.9951(11)	19.14(3)
β, °	95.0291(5)°	95.0132(7)	94.9623(5)	94.8670(10)	94.8131(5)	94.7601(7)	94.601(19)
Volume, Å^3^	3635.8(3)	3643.7(4)	3666.5(3)	3689.0(4)	3714.0(3)	3744.4(4)	3805(11)
*D*_calc_, g cm^−1^	1.421	1.418	1.409	1.401	1.391	1.380	1.358
μ, mm^−1^	0.390	0.389	0.387	0.384	0.382	0.378	0.372
*T*_min_	0.7456	0.4305	0.7456	0.4309	0.7456	0.7456	0.7456
*T*_max_	0.6942	0.3780	0.6971	0.3928	0.7059	0.6858	0.5867
Cr. max, mm	0.41	0.21	0.41	0.35	0.41	0.41	0.41
mid, mm	0.20	0.11	0.20	0.22	0.20	0.20	0.20
min, mm	0.17	0.10	0.17	0.15	0.17	0.17	0.17
Rfl. Measured	51759	41490	41636	38803	52563	42717	41322
Index ranges: *h*	−14, 14	−14, 14	−14, 14	−13, 13	−14, 14	−14, 14	−14, 14
*k*	−23, 23	−23, 23	−23, 23	−22, 22	−23, 23	−23, 23	−23, 23
*l*	−24, 24	−24, 24	−24, 24	−23, 23	−24, 24	−24, 24	−24, 24
θ limit, °	27.480	27.437	27.427	26.373	27.388	27.440	27.448
Unique data	8283	8298	8302	7522	8381	8517	8594
Restraints	34	64	46	170	62	0	0
Parameters	516	536	542	542	542	470	470
G.O.F. on *F*^2^	1.038	1.051	1.041	1.027	1.026	1.055	1.051
*R*_1_	0.0298	0.0361	0.0357	0.0326	0.0339	0.0467	0.0492
*wR*_2_ [*I* ≥ 2σ(*I*)]	0.0765	0.0800	0.0935	0.0853	0.0903	0.1216	0.1287
Largest pk, *e*Å^−3^	0.422	0.391	0.584	0.378	0.359	0.529	0.548
Largest hl, *e*Å^−3^	−0.297	−0.333	−0.281	−0.287	−0.289	−0.565	−0.497

Positional disorder was encountered in all crystals measured at all temperatures; broad descriptions of the disorder models are discussed under Section 2.1.2. Further details of these models follow. For the structure at 100 K, a difference map clearly shows the presence of a second component with the capping N9 atom rotated into the -ve *z* position along the molecular 2-fold axis. The occupancies refined to 0.92 and the disorder was then fixed at 90:10. The minor component was restrained to the average bond-distances of the main component. While it was possible to refine the four S atoms of the minor component anisotropically, doing so for the 5 N atoms required additional restraints that compromised the geometry, so these were left isotropic and refined to very reasonable displacement parameters. This disorder model was judged to be satisfactory, leaving only a single peak in the final difference map within the S,N cage (0.42 *e*/A^−3^), in a location indicative of S-N bonding density. At 120 K, a difference map clearly shows the presence of a second component with the capping N9 atom rotated into the site of N6 of the main component. The occupancies refined to 0.87 and the disorder was then fixed at 85:15. The minor component was restrained to the average geometry of the main component. Full anisotropic refinement was possible for this second component. This disorder model was judged to be satisfactory, leaving only one anion residual peak of 0.30 *e*/A^−3^ in the vicinity of S1. 

At 140 K, a difference map clearly shows the presence of a second component with the capping N9 atom rotated into the −ve *z* position along the molecular 2-fold axis. The occupancies refined to 0.68 and the disorder was first fixed at 70:30. The minor component was restrained to the average geometry of the main component. This model led to unacceptably high temperature factors for N6A. Adjusting the occupancy for N6A to correct for this optimized at 15%. The remaining intensity was then distributed to all other sites except N2 since N2 is the "open" side for the third component of the disorder. This model worked quite well and allows for full anisotropic refinement of the minor component. There are some peaks clustered around N9 (0.59 *e*/A^−3^) in the final difference map, suggesting that this model is not entirely correct. However, the overall model with 70:15:15 occupancies for main, ‘adjacent’ and ‘opposite’ is judged to be acceptable. For 173 K, the treatment follows the same course as at 140 K. The occupancies of the two-site ‘opposite’ model refined to 0.65 and the disorder was first fixed at 60:40 and checked by sequential isotropic refinements. This indicated the presence of a third positional component, which was modelled by raising the N9 and lowering the N6A occupancies. Upon anisotropic refinement, further adjustments were necessary to obtain reasonable displacement ellipsoids, and the final model adopted has N9 at 80% and N6A at 20%. Thus, the three-component positional disorder fits to a 60:20:20 ratio and is judged very successful as there are no significant residual peaks in the final difference map at or near the anion.

The procedure used for the 200 K data set follows the same approach as that used at 173 K. An excellent fit is obtained with N9 at 75%, N6A at 20% and N9A at 48%. This corresponds to a 3-site disorder model in approximate ratio 0.57:0.25:0.18. This model results in all peaks in the final difference map corresponding to bonding e.d. However, at the highest temperatures of 240 and 280 K, this approach did not work. After considerable trial and error, it was concluded that the best model was one with equal populations of all six potential N atom sites, each at 5/6th occupancy. This model no longer supports individual component coordinates so all atom positions are also averaged. This model works well crystallographically, although it obscures the known differences in bond distances as observed in the lower-temperature data sets.

Hybrid density functional theory calculations were undertaken with the B3LYP functional for S_4_N_5_^−^ using the crystal structure coordinates as a starting point and optimizing with a relatively small basis set (6-31G(d)). From this starting point, full geometry optimization and frequency calculations were performed with the 6-311++G(3df) basis set, with and without *2mm* symmetry. The calculations converged and reported zero imaginary frequencies. All calculations were performed using Gaussian03W and the results visualized with the aid of GaussView 5.0.9 [62].

## 4. Conclusions

Binary sulfur nitrides such as the S_4_N_5_^−^ ion are fundamental species which remain of current interest [63], and their structures typically make it into textbooks. It is therefore gratifying to be able to report for the first time a high-resolution crystal structure for this anion. The chemical proclivity of this species towards explosive decomposition must have dampened past efforts to find better salts to employ in diffraction studies and to our knowledge none have been found that are as good as PPN^+^ for safe handling. Thus, despite the challenges that the rather large cavities in the PPN^+^ salt provides and the deleterious effects of positional disorder on the metric parameters for the anion, this study shows that the PPN^+^ salt provides a very delicate surrounding, with just enough environmental influence to orient the anion at low temperature. Thus the 100 K and 120 K structure determinations from the dominant anion components are of excellent quality. 

A thorough analysis of both the disorder phenomenon and the supramolecular interactions within the crystal lattice provides evidence that the behavior of [PPN^+^][S_4_N_5_^−^]∙CH_3_CN is not random. Non-classical H-bonds interact with the anion in a well-defined manner. However, the results to date do not allow for a clear discrimination between possible “rotational jump” or 1,3-nitrogen shift mechanisms for the internal reorganization of the anion that is observed by crystallography.

In conjunction with the first high-resolution diffraction study, this report provides an updated computational structure by modern DFT methods. These are in excellent agreement with the best crystal structure geometry. Moreover, the calculated vibrational spectrum is found to match well with literature reports and allows for an assignment of the major infra-red spectral bands.

## Supplementary Materials

Full details of the X-ray structure determinations in electronic crystallographic information file (CIF) format. (This file also provides coordinates for Tl_2_[S_3_N_3_][S_4_N_5_] and the DFT-calculated geometry.) Source table of data for the graph in Figure 6. Lists of coordinates from DFT optimizations. They can be accessed at: http://www.mdpi.com/1420-3049/19/2/1956/s1.
