# Supplementary material for: A Variable Temperature X-ray Diffraction Investigation of [PPN+][S4N5−]: Supramolecular Interactions Governing an Order/Disorder Transformation and the First High Resolution X-ray Structure of the Anion
Source: Molecules. 2014 Feb 12;19(2):1956–75. doi: 10.3390/molecules19021956 (PMC6271633; doi:10.3390/molecules19021956)
Supplement: Supplementary file 1 [file molecules-19-01956-s001.zip › molecules-49065-supporting information.pdf]

Supporting Information

Source table of data for the graph in Figure 5	Table S1
Lists of coordinates from DFT optimizations	Table S2,3

Table S1. Data for the analysis of unit cell and cavity "host" volumes.

**Temp**	**Unit Cell Vol**	**Anion vol**	**Anion Vol %**	**Cavity Vol**	**Cavity Vol %**	**Solv Vol**	**Solv Vol %**
100	3635.8	556.76	15.3%	829.32	22.8%	272.56	7.5%
120	3643.73	562.52	15.4%	839.56	23.0%	277.04	7.6%
140	3666.47	567.85	15.5%	864.99	23.6%	297.14	8.1%
173	3689.03	592.88	16.1%	885.52	24.0%	292.64	7.9%
200	3714.03	602.28	16.2%	913.2	24.6%	310.92	8.4%
240	3744.39	639.71	17.1%	971.46	25.9%	331.75	8.9%
280	3805.51	665.18	17.5%	1012.8	26.6%	347.62	9.1%
Total incr	169.71	108.42	2.17%	183.48	3.80%	75.06	1.64%

Table S2. List of coordinates from DFT optimizations of $[S_4N_5]^-$ in B3LYP/6-311++G(3df) – with symmetry restriction to C_{2v}.

Total energy = -1866.63938873 Hartree. Stoichiometry N5S4(1-)

Framework group C2V[C2(N),SGV(S2),SGV'(S2),X(N4)]

Deg. of freedom	7		
Full point group	C2V		
Largest Abelian subgroup	C2V	NOp	4
Largest concise Abelian subgroup	C2V	NOp	4

			**Coordinates (Angstroms)**		
**Center Number**	**Atomic Number**	**Atomic Type**	**X**	**Y**	**Z**
2	16	0	−1.42135	0	−1.0644
3	16	0	0	−1.39569	0.856442
4	16	0	1.421352	0	−1.0644
5	7	0	−1.29304	−1.35735	−0.19578
6	7	0	1.293039	−1.35735	−0.19578
7	7	0	1.293039	1.357345	−0.19578
8	7	0	−1.29304	1.357345	−0.19578
9	7	0	0	0	1.733792

Table S3. List of coordinates from DFT optimizations of $[S_4N_5]^-$ in B3LYP/6-311++G(3df) – without symmetry restriction.

Total energy = −1866.63939127 Hartree. Stoichiometry N5S4(1-)

Framework group C2V[C2(N),SGV(S2),SGV'(S2),X(N4)]

Deg. of freedom	21		
Full point group	C1		
Largest Abelian subgroup	C1	NOp	1
Largest concise Abelian subgroup	C1	NOp	1

			Coordinates (Angstroms)		
**Center Number**	**Atomic Number**	**Atomic Type**	**X**	**Y**	**Z**
1	16	0	1.394859	−0.047679	0.856588
2	16	0	0.049941	1.420022	−1.064534
3	16	0	−1.39483	0.047656	0.856623
4	16	0	−0.04994	−1.419998	−1.064585
5	7	0	−1.31122	1.339442	−0.196525
6	7	0	−1.40222	−1.245278	−0.195186
7	7	0	1.311208	−1.33945	−0.196478
8	7	0	1.402222	1.245317	−0.195028
